# Metal and Polymer Based Composites Manufactured Using Additive Manufacturing—A Brief Review

**DOI:** 10.3390/polym15112564

**Published:** 2023-06-02

**Authors:** Sundarakannan Rajendran, Geetha Palani, Arunprasath Kanakaraj, Vigneshwaran Shanmugam, Arumugaprabu Veerasimman, Szymon Gądek, Kinga Korniejenko, Uthayakumar Marimuthu

**Affiliations:** 1 Institute of Agricultural Engineering, Saveetha School of Engineering, Saveetha Institute of Medical and Technical Sciences, Chennai 602105, India; sundarakannan.r@gmail.com (S.R.); kesangee@gmail.com (G.P.); 2 Department of Mechanical Engineering, PSN College of Engineering and Technology, Tirunelveli 627152, India; aruncmr12@gmail.com; 3 Instituteof Mechanical Engineering, Saveetha School of Engineering, Saveetha Institute of Medical and Technical Sciences, Chennai 602105, India; s.vigneshwaren@gmail.com; 4 Faculty of Mechanical Engineering, Kalasalingam Academy of Research and Education, Krishnankoil 626126, India; v.arumugaprabu@klu.ac.in; 5 Faculty of Materials Engineering and Physics, Cracow University of Technology, Al. Jana Pawła II 37, 31-864 Kraków, Poland; szymon.gadek@pk.edu.pl

**Keywords:** 3D printing, additive manufacturing, fiber composites, polymer, metal, mechanical properties

## Abstract

This review examines the mechanical performance of metal- and polymer-based composites fabricated using additive manufacturing (AM) techniques. Composite materials have significantly influenced various industries due to their exceptional reliability and effectiveness. As technology advances, new types of composite reinforcements, such as novel chemical-based and bio-based, and new fabrication techniques are utilized to develop high-performance composite materials. AM, a widely popular concept poised to shape the development of Industry 4.0, is also being utilized in the production of composite materials. Comparing AM-based manufacturing processes to traditional methods reveals significant variations in the performance of the resulting composites. The primary objective of this review is to offer a comprehensive understanding of metal- and polymer-based composites and their applications in diverse fields. Further on this review delves into the intricate details of metal- and polymer-based composites, shedding light on their mechanical performance and exploring the various industries and sectors where they find utility.

## 1. Introduction

Additive manufacturing (AM) has emerged as an advanced and innovative technique within the manufacturing industry. This technique, is also known as 3D printing, it has proven to be highly effective in utilizing reinforcements such as fillers and fibers in the fabrication of polymers and metals. By employing a layer-by-layer material deposition approach, AM enables the creation of composites, while conventional methods relying on subtractive manufacturing are used for comparable product development [1]. The utilization of AM offers several significant advantages, including cost-effectiveness and the ability to design and fabricate complex structures with precision and high quality. These advantages have positioned AM as a preferred technique, particularly in the aerospace and automotive sectors, where intricate and accurate products are in high demand. The development of 3D-printed composites has witnessed substantial progress over the last decade, and it is expected that these materials will play a pivotal role in revolutionizing diverse industries in the future [2,3].

The utilization of AM technology is widespread in aerospace, electrical, and biomedical applications [4]. However, in areas such as architecture and the construction industry, its implementation is still limited [5,6]. One notable advantage of AM is its ability to reduce material waste and lead times, offering a flexible manufacturing approach. Incorporating waste or natural fibers as additives hold great promise for enhancing the environmental impact of composite materials in these fields [7]. It is important to highlight that manufacturing natural fiber-reinforced composites using the AM process presents certain challenges. Factors such as fiber interactions, weight percentage, type, orientation, and length need to be carefully considered during composite material development. Nonetheless, AM serves as an excellent method for producing innovative and complex composite materials [7].

This review aims to provide a comprehensive summary of the current state of the art in the mechanical performance of metal and polymer-based composite materials fabricated through AM. The review is based on an extensive examination of scientific literature published within the last five years, utilizing reputable sources such as Science Direct, Scopus, and Google Scholar. By analyzing the latest trends and research findings, this review highlights the advancements and potential applications of composite materials in the context of AM. This article serves as a comprehensive guide for researchers and professionals who seek to gain an understanding of the recent developments, challenges, and opportunities in the field of AM-based composite materials.

## 2. Fabrication of Composite Materials

Composite materials produced using additive manufacturing (AM) techniques have undergone significant advancements throughout the years. Initially, AM methods such as stereolithography (SLA) and Fused Deposition Modeling (FDM) were utilized to create plastic prototypes. Subsequently, there was a development towards developing polymers and metal matrix-based composites using AM, primarily due to their ability to manufacture intricately shaped structures [8]. Further to enhance performance, high-performance composites were developed using carbon fiber and graphene, which exhibit improved thermal and electrical properties. Moreover, the AM concept extended to lightweight structural applications through the use of glass particles as reinforcement, combined with synthetic foam [9]. Among AM methods, FDM is particularly well-suited for fabricating polymer-based composites. Thermoplastic filaments are commonly employed in the FDM process. This method offers advantages such as low cost and the ability to vary chemical and mechanical properties. In addition to FDM, other familiar AM techniques used for manufacturing polymer-based composites include sheet lamination, material extrusion, photopolymerization, and powder bed fusion. Photopolymerization provides finer resolution compared to other methods. Material extrusion, on the other hand, is the simplest and most cost-effective method, making fabrication easier.

Metal composites-based additive manufacturing (AM) techniques are a specialized subset of additive manufacturing that focuses on fabricating components using metal matrix composites. These techniques involve the incorporation of reinforcement materials, such as ceramic or carbon fibers, within a metallic matrix. By combining the unique properties of different materials, metal composites offer enhanced mechanical strength, improved thermal properties, and increased lightweight capabilities [10]. Metal composites-based AM techniques, such as powder bed fusion (PBF) and directed energy deposition (DED), enable the production of complex and high-performance metal composite parts with precise control over material composition and fiber distribution. Several notable metal AM techniques are:

Powder Bed Fusion (PBF): PBF includes selective laser melting (SLM) and electron beam melting (EBM). In SLM, a high-powered laser selectively fuses metal powder particles layer by layer to create the desired metal part. EBM, on the other hand, uses an electron beam to melt the metal powder and form the object [11]. PBF techniques offer high precision, intricate geometries, and excellent material properties.

Directed Energy Deposition (DED): DED techniques, such as laser metal deposition (LMD) and electron beam freeform fabrication (EBF3), involve depositing molten metal layer by layer onto a substrate or previous layers. This method is particularly useful for repairing or adding features to existing parts, as well as fabricating large-scale components [12].

Binder Jetting (BJ): Binder jetting utilizes a liquid binder to selectively bond metal powder particles together. The printed part is then subjected to a secondary process, such as sintering or infiltrating, to achieve the desired mechanical properties. BJ is known for its high productivity and suitability for producing complex geometries [13,14].

Wire Arc Additive Manufacturing (WAAM): WAAM involves melting and depositing a metal wire using an electric arc. This technique is cost-effective and can be used for large-scale manufacturing. WAAM is commonly used in the aerospace, automotive, and maritime industries [15].

Ultrasonic Additive Manufacturing (UAM): UAM employs ultrasonic vibrations to join layers of metal foils together. This technique allows for the integration of dissimilar metals and can be used for fabricating lightweight structures [16].

These metal-based AM techniques offer numerous advantages, including design freedom, reduced material waste, faster prototyping, and the ability to create complex and customized metal parts. They find applications in various industries, including aerospace, automotive, medical, and tooling, among others [17]. Continuous research and development efforts in metal AM are further advancing the capabilities and expanding the possibilities of metal-based additive manufacturing. 

## 3. Polymer-Based Composite Materials—Performance

The motivation for producing polymer-based materials through additive manufacturing (AM) is to enhance their properties and expand their applications across various sectors. Natural fibers such as wool, hemp, flax, kenaf, and vegetable fibers have been successfully utilized as replacements for artificial fibers in composite manufacturing using AM. AM is a manufacturing process that allows for the creation of complex shapes with minimal material waste and time. Different types of polymers, including thermoplastics, liquid polymers, and reactive polymers, are used in AM, with recent advancements focusing on incorporating fillers such as nanotubes, carbon fibers, nanofibers, nanoparticles, and synthetic fibers into polymeric products [18]. The production of lightweight polymeric products presents challenges in engineering industries, and the development of AM techniques has alleviated some of these burdens in composite manufacturing [19]. Notably, AM-based technology has demonstrated superior performance, particularly in Fiber-Reinforced Polymer (FRP) materials, resulting in the production of high-performance structural components [20].

**Figure 1:**
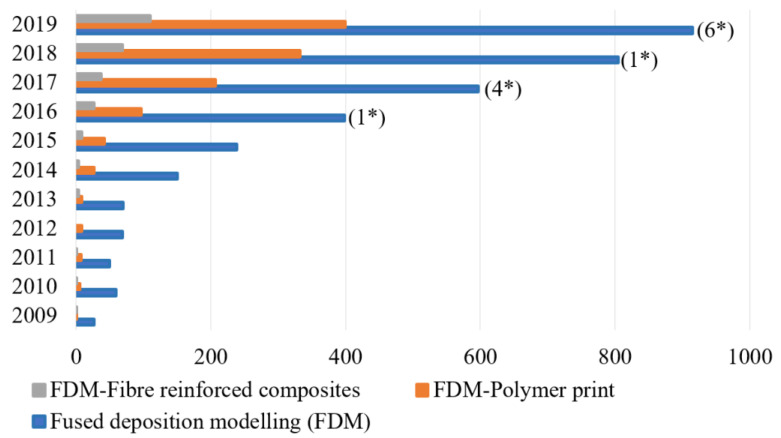
Number of publications on FDM, FDM-polymer prints and FDM-fiber-reinforced composites from 2009 to 2019. The numbers with * within the brackets indicate the number of review papers on FDM-fiber-reinforced composites. (Reprinted with permission from [22]).

Fused Deposition Modeling (FDM) is a widely adopted additive manufacturing technique employed for the production of polymeric components. This method involves the layer-by-layer deposition of materials, resulting in well-structured parts with minimal waste generation in the form of chips [21]. The utilization of additive manufacturing allows for the fabrication of complex shapes while reducing chip formation and waste production. The number of publications on FDM, FDM-polymer prints and FDM-fiber-reinforced composites from 2009 to 2019 is shown in Figure 1. The numbers within the brackets indicate the number of review papers on FDM-fiber-reinforced composites. FDM-based polymeric models exhibit superior mechanical properties compared to other additive manufacturing processes.

**Figure 2:**
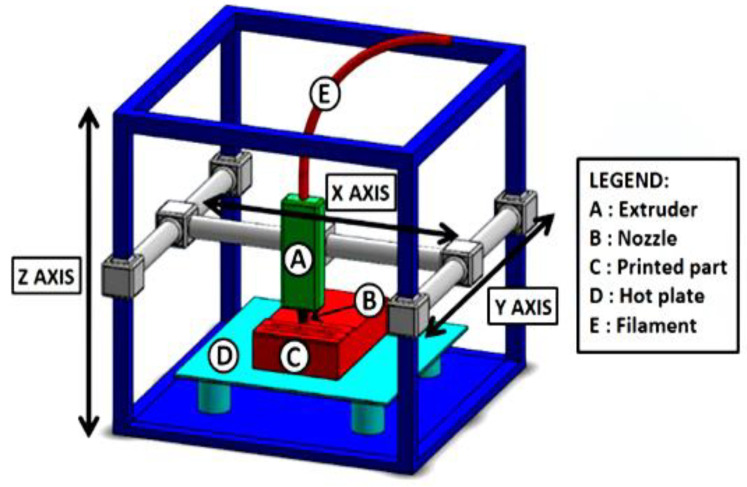
Typical FDM setup (reprinted with permission from [26]).

The rectilinear filling of PLA with honeycomb structured fillers showed 15% higher mechanical properties, due to its better-chosen filling pattern, print speed, layer thickness and nozzle diameter [23,24]. Three-dimensional printed polylactic acid (PLA) composite parts with carbon fiber (CF) processed using the FDM technique have enhanced tensile, flexural, and interlaminar shear. The PLA-CF combination showed a ca.47% increase in tensile strength compared to the pure form of CF and PLA [25]. Similarly, the same results were observed for the same combinations as ca.90% in flexural strength and ca.72% for interlaminar shear than in the simple forms of CF and PLA. Figure 2 shows a typical FDM process. Low strain in PLA was increased by adding carbon fiber which led to high-performance PLA-based polymeric composite.

The ternary carbon black (Bi_2_Te_3_) combined with PLA-based polymeric composite produced using a stereolithography technique and their thermoelectric (TE) properties has recently been investigated [27,28,29,30]. Polymeric components manufactured from additive manufacturing techniques have better thermoelectric properties than other traditional manufacturing techniques.

The physical properties of the polymer ensure the compatibility of the polymer with reinforcements in achieving better properties. Polymeric materials manufactured through AM technology usually have better applications in making flexible, corrosion-free, and optically transparent parts [31]. FDM material is available in filament form, liquid resin for Selective Laser Sintering(SLS) and SLA, and 3D-Scanning, 3D Printing, 3D Modeling, 3D Visualization, and VFX materials in the form of powder. The printing temperature of these materials shows better results between 120 °C and 450 °C [32].

**Figure 3:**
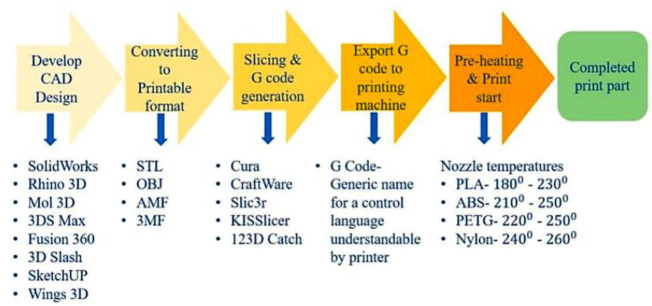
The FDM process flow diagram shows various software types employed in the suggested nozzle temperature and process for fewer materials (Reprinted with permission from [22]).

Vat photopolymerization (VP) is one of the AM techniques in which the polymerization process occurs for photosensitive resins using ultraviolet and visible light. The VP additive manufacturing technique is well-known for part assembly and resolution of parts. The thermo-mechanical behavior of the polymer components produced by VP techniques results in better performance [33]. The rapid heating and cooling of polymers increase the functional behavior of the polymeric composite [34,35]. Figure 3 presents the FDM process flowchart, illustrating the various types of software used in the recommended nozzle temperature and process for a few materials.

Yangetal. [36] studied the volumetric energy density (Ev), mechanical strength, and densification behavior of the bioglass polymeric composite. On the surface of the composite, the presence of a larger amount of liquid was found, which was responsible for the discontinuous surface and poor lifetime of the polymeric composite [37,38,39]. The component was fabricated with the lithography technique and has a smooth surface, and has a high densification of ca.185 J/mm^3^ Ev value and enhanced mechanical strength of about ca.153 MPa with very good densification [40].

**Figure 4:**
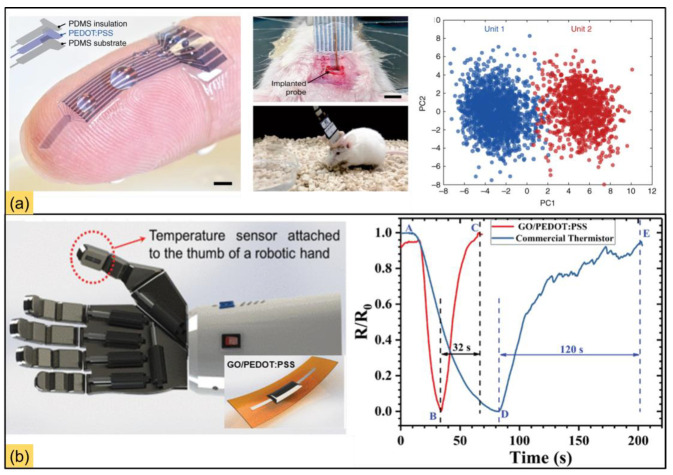
CPs 3D printing to be employed as sensors. (**a**) PEDOT:PSS electrodes printed with high resolution by extrusion-based printing and implanted in the cranium of mice for electrophysiological recordings. (**b**) GO and temperature sensor based on PEDOT:PSS inserted in a robot interface, which allows physical interaction detection with objects in the real world, and improves the commercially available thermistor. (Reprinted with permission from [43,44]).

High-performance polymers (HPPs) have a wide range of applications that can be fabricated using AM techniques [41]. The demand for advanced polymer fabrication using AM techniques for HPPs creates a platform for innovative products to be used in material science, mechanical engineering, and multidisciplinary approach fields. Digital light processing is the strong additive manufacturing process used to produce higher liquid resins such as polyimide, bismaleimide, and cyanoester. In Figure 4, various 3D-printed forms of CPs intended for use as sensors are depicted in detail. Thermoplastics such as poly (ether imide) (PEI) and polyether ether ketone (PEEK), with high-performance properties, are utilized as filament materials for high-temperature applications over 1770° [42].

The strength of composites fabricated through additive manufacturing (AM) can be negatively affected by the agglomeration of fiber reinforcements at high-volume fractions. To address this issue, the integrated vibration auger extension system (VIAES) technique has been developed. VIAES is a fabrication method specifically designed for the preparation of fiber-reinforced thermoset composites, offering the potential to achieve high fiber volume fractions in polymers. Composites produced using the VIAES technique demonstrated a flexural strength of 401 MPa, compression strength of 673 MPa, and flexural stiffness of 53 GPa, with a fiber volume fraction of 46%. Notably, a strong adhesion between carbon fibers and epoxy resin was observed, leading to enhanced mechanical strength in these composites [45].

Various printing factors also influence the performance of printed parts. Laser power, particle size, build volume, and printing temperature are among the factors that affect the accuracy of the final product. Precision, particularly in the selective laser sintering (SLS) method, is crucial as the mechanical strength of the composite relies on the build volume and printing speed. This advantage enables the production of complex structures with improved mechanical properties compared to the FDM method [46]. The high fluidity and thin layer of powder prints, combined with the quality of raw materials, contribute to the development of superior surface finish and highest-resolution polymeric materials [47]. A summary of the general characteristics of polymer-based additive manufacturing techniques is shown in Table 1.

## 4. Performance of Metal-Based Composite Materials

Metal-based functional graded materials play a crucial role in achieving high-quality welds and joints, particularly when dealing with complex shapes. However, there are various challenges associated with these materials, including their chemical, metallurgical, and thermal properties. The particular concern is the thermal property, as it presents a barrier for the material coating to withstand temperatures exceeding 1000 degrees Celsius and thicknesses greater than 10 mm [48]. Fortunately, additive manufacturing (AM) provides a promising solution for producing top-notch products that involve metal joining processes. Metals offer excellent functional properties, including lightweight structures and superior resistance to high temperatures and corrosive environments. By leveraging AM techniques, the fabrication of metal-based functional graded materials can be optimized to meet the demanding requirements of various applications [49].

When considering the strengths and weaknesses of fiber-based additive manufacturing (AM) products, the combination of fibers with metals yields favorable outcomes. Particularly for high-temperature applications, the impregnation between the fiber and the metal enhances bonding compared to using clean metal and fibers alone [50]. The mechanical performance of these composite materials is also significantly improved, with a 65% increase compared to fiber–fiber and metal–metal combinations. These advancements propel AM technology to the next level, enabling the production of more substantial polymer–metal parts [51].

In a study by Dong et al., the performance of titanium (Ti) and titanium alloys (TiB) manufactured through selective laser sintering was discussed. Among the metal powder-based composites, Ti-TiB stands out as a cost-effective option with excellent mechanical properties achieved by optimizing the process parameters. By incorporating TiB_2_ in varying weight percentages, ranging from 0.5 wt% to 2 wt%, into titanium, notable improvements were observed. Notably, Ti-0.5 wt% of TiB2 exhibited an enhanced tensile strength of 1813 MPa and a microhardness of HV 412 [52,53].

Cevik and Kam conducted a study on composite filaments using the fused deposition modeling (FDM) method to examine their mechanical properties [54]. Through the addition of polymers with metals, improved mechanical properties such as impact strength, hardness, tensile strength, elastic modulus, fatigue strength, and yield strength were observed. In FDM, the selection of additives and their proportions play a crucial role in achieving desirable mechanical performance [55,56]. Furthermore, it was found that increasing the layer thickness and the diameter of the nozzle by 15% in the fill patterns of finished parts contributed to better mechanical properties [57].

In the context of composite materials, fatigue refers to the cyclic loading they experience during their operational lifespan. Fiber materials are commonly used in the manufacturing of leaf springs, but metal-based composites have demonstrated superior performance compared to fiber composites [58,59]. Metal-based composite laminates produced using AM techniques have shown better results. When compared to boron/epoxy and Kevlar/epoxy composite leaf springs, the metal-based composite exhibited 44% less stress and 38% higher stiffness [60].

The 40% HDPE/GME composite exhibits excellent adhesion during printing, and finite element analysis (FEA) of thermo-mechanical processes reveals a low distribution of thermal stress across different layers in the prints. This suggests that AM techniques offer improved strength compared to conventional methods in the manufacturing of metal composites [61,62].

To enhance the bonding between PLA layers, aluminum (Al) spray was deposited between the PLA layers. The incorporation of metal was varied at percentages of 40%, 70%, and 100%, while the bed temperature was adjusted between 60, 80, and 100 degrees. The highest bed temperature of 100 degrees facilitates better fusion and results in stronger bonding between layers. Moreover, higher temperatures increased the crystallinity of PLA in the composite, leading to enhanced heat transfer between layers [63].

**Figure 5:**
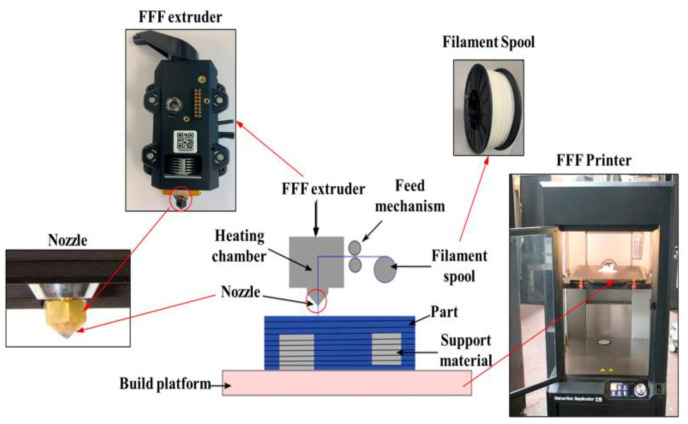
Overview of Fused filament fabrication printer process (reprinted with permission from [66]).

A new class of materials known as metallic cellular solids possesses unique mechanical properties. Figure 5 illustrates a schematic representation of the fused filament fabrication (FFF) process. Fused filament fabrication was utilized to create lattice-structured patterns using PLA plaster and aluminum [64]. The honeycomb structure has a struct angle of zero, owing to deviations in the moment of inertia and cross-sectional area. Combining PLA with the aluminum model yields a well-defined metallic cellular lattice with high tolerances. Additionally, AM serves as a cost-effective alternative to conventional methods for fabricating metal composites [65].

Wang et al. [67] studied the fracture and ductile failure mode behavior of aluminum (Al) prepared to produce composite additive manufacturing (CFAM) using cold spray-friction stir processing. There was no formation of defects such as pores and cracks noticed in the CFAM-manufactured composites. The layers of the composite were divided into three zones, namely top, bottom, and middle, the grain size distribution in various zones varies with the microstructure by the top zone (0.3–25 µm), middle (0.3–22 µm), and bottom (0.3–18 µm) which clearly define the perfect bonding and recrystallization of the AM samples.

Another important problem is the energy transfer through fluid channels, during AM technique usage. Compared to lightweight applications, the fluid transmitted through channels has more energy transfer and strength. The advantage of heat transfer during AM techniques is very essential to producing a higher energy conversion between the layers of metal laminates. The effective design of components and their performance is purely dependent on the channel flow of the multi-material and multi-process in the AM technique [68].

The high-performance integrated system is used in the selective laser sintering (SLS) process to enhance the mechanical properties of hybrid composites. When the microstructure of metallic components on the layer surface is refined, the surface micro-hardness is improved. It reduces the residual tensile and compressive stress between the layers of the hybrid composite. Using this technique, a reduction in residual stress is observed at approximately 310 MPa and 396 MPa, respectively with a depth of 0.9 mm.

The low- and high-speed composite impact resistance manufactured through the AM technique shows improved results. The multi-wall of carbon and aluminum absorbs very low energy during the time of low-velocity impact event, happening about 18.03% compared to other samples of carbon/aluminum. In the time of high velocity, an event that occurs for the same (carbon/aluminum) very low amount of velocity is observed between 84 m/s to 150 m/s. The particle and settlement of the metal on the fiber produce a superior property for the composite made through AM techniques [69]. 

The thermal expansion of the aluminum/titanium composite is measured for the composite produced through 3D printing. The alternative arrangement of increased titanium layers in the composite has better thermal expansion. The unique combination in the time of composite production acts as a potential barrier in transmitting the heat between the layers. The measured thermal expansion is around 17.1 × 10^−6^ K^−1^ and this is a control expansion that shows the accuracy of the composite model that gives greater thermal stability [70]. Table 2 represents the characteristics of metal-based AM techniques.

## 5. Applications

The technological advancement in the business area leads to the incorporation of AM by industries to produce a component. In the industry part development concern, the AM technique saves more time, offers good dimensional stability, and limits the waste of material during the time of manufacturing [71]. Nowadays, the AM fabrication technique is one of the wide ranges of methodologies used to produce most of the engineering components. In that sense, sensors are one of the products which are mainly fabricated through this technique. Sensors for engineering applications such as mechanical, temperature, particle, and tactile sensors are manufactured through this technique. Additionally, for biomedical applications, bio-molecular, microbial, cell-based, and bionic sensors were manufactured. These sensors are manufactured by 3D printing with PLA, graphite, and thermoplastic polyurethane as printed material [72]. 

**Figure 6:**
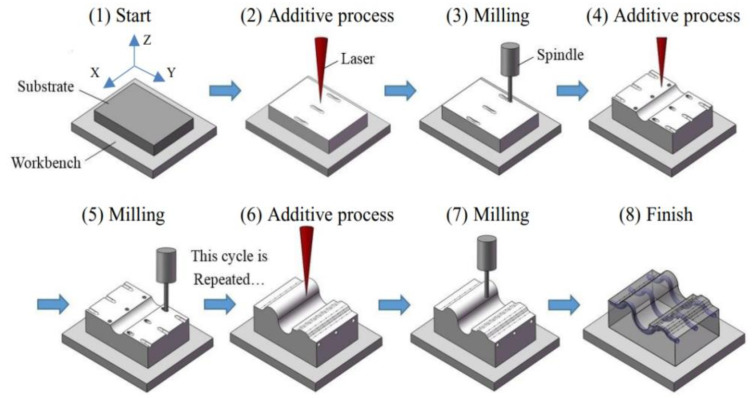
3D printing, additive manufacturing, and subtractive manufacturing (reprinted with permission from [75]).

AM technology extended in the area of dental implants, bone mesh with titanium (Ti), and cobalt–chromium (Co-Cr). The mechanical and micro-structural properties of chromium and cast steel have improved properties, during the fabrication using SLS, SLM, and PBF AM techniques [73]. Seismic isolation bearings are one of the sensational discoveries of the AM technique, which is predominately used in the automotive industry. This is manufactured using a binder jet printer (BJM), with 190 m quartz sand of 190 µm. Very good compressive and tensile strength is noted for these materials, with better curing at room temperature [74]. The novel drug discovery system involves this AM technique for producing dosed drugs like tablets, pills, and capsules for various diseases. The advantages of 3D printing over subtractive manufacturing are represented in Figure 6. The pharmaceutical and medicine industries have widely used AM techniques, such as FDM, binder printing, SLA, and SLS for compatible packaging purposes. 

Few polymers (carbon, nylon, ABS) and metals (copper and nickel) have good optical properties and are chemically stable in various climatic conditions. Products such as dolls, reflective mirrors, and some pictorial structures are made with these materials fabricated through AM techniques. The transparent photo-resisting property of these materials attracts people to view the products as more colorful [76]. Using 3D-printing AM technology, instruments such as custom trays, surgical guides, provisional restorations, and implant casts were manufactured. It is a virtual-based scanner model that significantly improves the accuracy of the implant sleeve. By using materials such as VisiJet M3 Stone plast and dental SG resin, most dental models are produced, which are used for the long-life application of the products [43]. In the automotive industry, owing to the less weight of components, most OEMs prefer AM-based products for metal replacement, and this type of digital platform helps to improve the sustainability of products and satisfy the needs of the customers [77].

In the construction sectors, concrete printing, free foam, powder bed, and contour crafting are some of the methods used to manufacture concrete, sand, and cement through the AM technique. Additive manufacturing produced better layer adhesion and build ability for finished constructive products [78]. Three-dimensional printing, SLM, FDM, and SLA are the highly necessary techniques that are used in the electrochemical process for energy transformation. The electrodes are fabricated using the AM technique which also acts as an insulator and keeps the chemical reactions under control [79].

Some of the thermoplastic materials are becoming cheaper and are used regularly for producing textiles, using 3D-printing technology. Using Netfabb: 3D printing software, the difficulty in the geometry of the textile was resolved and fine quality was achieved through optimization and ankle braces of textiles were produced [80,81].

The customization of AM technology is mostly preferred to fabricate bones, tissues, and human organs. In addition, it plays an important role in providing treatments for valvular heart disease, heart disease, electrophysiology, physiology, cardiac masses, and diagnostic testing. AM technology also provides a promising application to print liver tissue and cells to fulfill customization functions [82].

### 5.1. 3D-Printed Rocket Components

3D printing, especially with metals, is employed in rocket production. Engineers can reinvent the rocket parts design and produce them in a shorter amount of time due to advances in technology [83].

#### Spotlight: Airbus

**Figure 7:**
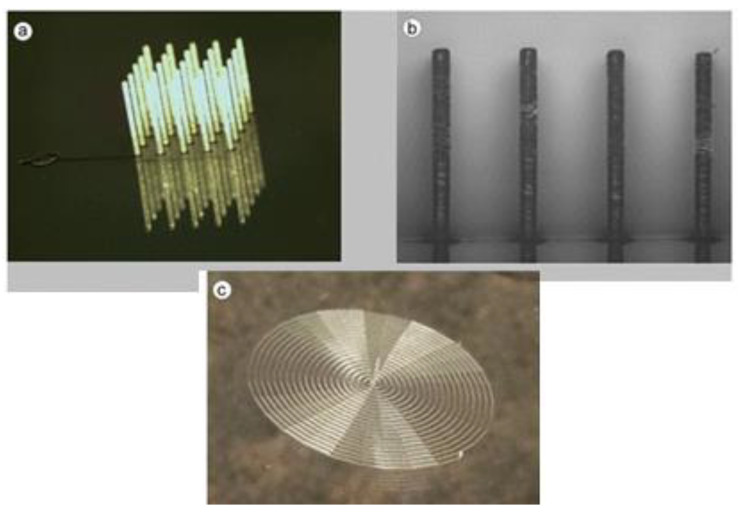
High aspect ratio 3D structures produced by an aerosol jet (**a**,**b**) pillar structures of the array (**c**) spiral structure and (Reprinted with permission from [77]).

In the aerospace industry, 3D-printed plastic components, such as those used in aeroplane cabins, can be of great benefit. Acrylic resin aerosol jet printing with simultaneous UV LED curing yields high aspect ratio 3D objects, as seen in Figure 7. At some point, the interior of a commercial plane may need to be renovated, which may involve replacing things like wall panels. Generally speaking, customization necessitates small-batch production of parts. We also require fast turnaround times. Airbus has used 3D printing FDM to build components with complicated characteristics, such as lattice structures, at no additional cost to manufacture [84]. The final result is 15% lighter spacer panels than panels made with conventional procedures, which helps reduce the overall weight of the aircraft.

### 5.2. 3D Printing during COVID-19

**Figure 8:**
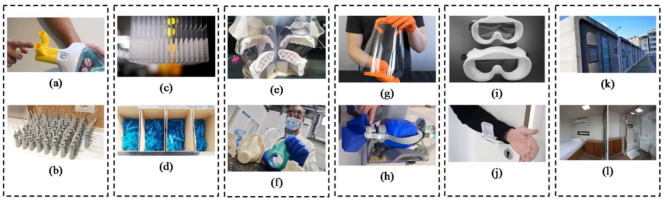
3D printing during COVID-19 outspread—Application overview. (**a**) Valves used to convert the snorkeling face masks into ventilators (**b**) Valves for respiratory devices (**c**) nasopharyngeal swabs (**d**) syringes (**e**) medical manikins for swabs (**f**) Silicon masks (**g**) protective face shields (**h**) emergency respiratory equipment (**i**) safety goggles (**j**) contact-free door handles (**k**) isolation wards (**l**) isolation houses equipped with a bed, shower, and toilet. (Reprinted with permission from [75]).

Globally, for medical personnel and patients, 3D printing technology is frequently utilized to fabricate PPE. During the COVID-19 pandemic, the private sector and individuals used 3D printers to create protective safety goggles, masks, contact-free door knobs, manikins, respirators, and ventilator pieces for healthcare personnel. Several companies have begun 3D printing safety eyewear and miniature quarantine enclosures for hospitals [85]. The Purdue University faculty and graduate students redesign and print the intricate components of face shields, safety glasses, and ventilators. Automobile manufacturers such as General Motors (GM), and Tesla began to play their role in the fight against the pandemic by promptly prototyping the PPE parts and ventilators to considerably enhance the availability of equipment to medical professionals and the public [86,87]. Figure 8 represents additional innovative contributions of 3DP to the fight against the pandemic, including nasopharyngeal swabs for preventive/diagnostic face syringes, shields, ventilator valves, testing, medical gloves, test tubes, and connections.

### 5.3. Digital Dentistry, Medical Modelling, and Prosthetics in AM Process

The consideration of design strategies, techniques, and materials allows the manufacture of customized 3D-printed parts, which is very advantageous for the medical sector. This technology is being applied to use in the medical field, including a spinal fusion cage, a dental model, a prosthetic hand, PPE, a sacral surgical planning model, and tiny needles for the administration of drugs [88,89,90,91,92].

**Figure 9:**
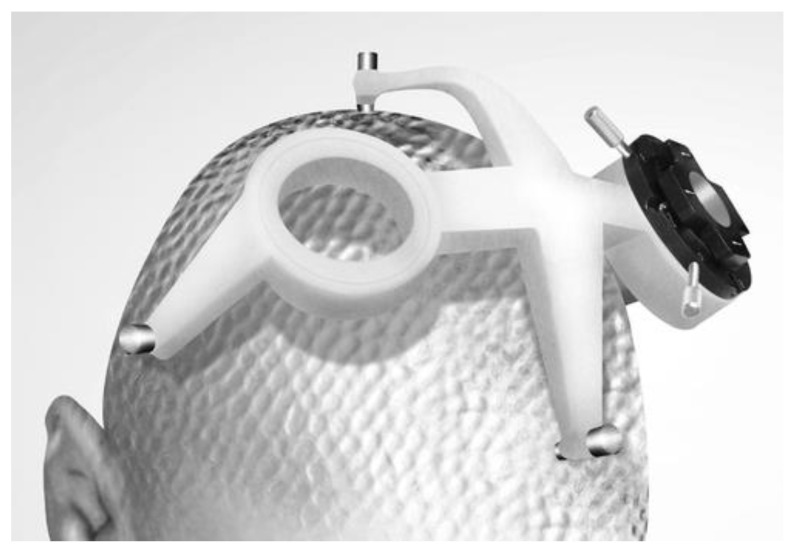
SLS with PA-12 powder manufactured customized neurosurgical guide (Reprinted with permission from [94]).

Patient-specific hearing aid cases made by Phonak and Material are one of the first examples of how AM has been used successfully not just for prototyping but also for creating things [93]. The 3D scanning or molding of the patient’s ear is the first step in making the polymeric casing. This creates an STL file that can be used by AM to make the casing. In the last 20 years, both the hardware and software used for 3D medical imaging (using magnetic resonance imaging (MRI) and positron emission tomography (PET)) have come a long way. Combining these techniques with AM has led to the creation of patient-specific models that can stand on their own and be used to plan hip, knee, shoulder, or brain tumor surgeries. As an example, SLS has been utilized with polyamide to make customized medical field parts, such as the neurosurgical guide. The guide piece is made for each patient based on their MRI and CT scans, and it helps align a micro drive to record and place a deep brain stimulation lead. With photo-based AM of elastomeric photopolymers, clear and flexible models of a whole heart have been formed with connected inner chambers, blood vessel structures, and mitral valve. Figure 9 shows SLS with PA-12 powder, a neurosurgical guide that is made to order.

### 5.4. Utilizing Additive Manufacturing in the Food Industry

In 2001, a patent showing the 3D-designed cake AM described the first efforts to utilize additive manufacturing technology in the food processing industry. Additionally, experts assert that AM can be used to improve the nutrition sources previously rejected by consumers, such as the “insects au gratin” project. Economic reasons [95,96] indicate that this technique is unlikely to deliver the promise of resolving the world’s nutrition problems.

### 5.5. 4D Printing Method

Objects that are conventionally 3D printed keep their original forms and attributes throughout their entire product lifetimes. Stratasys, Inc. pioneered 4D printing and Skylar Tibbits of the MIT Self-Assembly Lab, which uses time as the 4D for 3D fabricated “smart” structures that change shape in programmable and predefined ways in response to external stimuli. This stimuli-responsive behavior, which is programmable through the selection of suitable printing materials and CAD, considerably widens the spectrum of traditional SMA, multifunctional materials, and systems smart materials [97]. One material retains its rigidity regardless of the presence of water, while the other (a hydrogel) swells significantly (>200%) in the presence of water. By carefully positioning expanding materials using 4D printing, it is feasible to build water-responsive joints that, when touched, morph by bending and folding into a wide range of CAD-programmed forms [98].

## 6. Future Scope

The future scope of AM in the production of composite materials plays a predominant role in determining very important properties of composites, such as fatigue behavior, especially the composites prepared using the FDM technique. The impact on the fatigue behavior of the composites is considerably varied when compared to the ordinary process which is more helpful in biomedical applications [99]. Further studies have been carried out on the thermal and mechanical characteristics of FDM fiber-based composites [100,101] and reported that the FDM is the cheapest and most time-consuming method but leads to improved strength in strength for a few cases that can be drastically changed by altering the printing parameters that creates huge future scope for further research. The fire resistance properties of 3D-printed composites were also reported [102], which is another potential to explore more in research by varying the printing parameters. Another interesting review focusing on the recycling and reuse of AM materials [103] inferred the fact that there are huge potential huge opportunities for recycled polymers to be utilized in the development of AM products.

**Figure 10:**
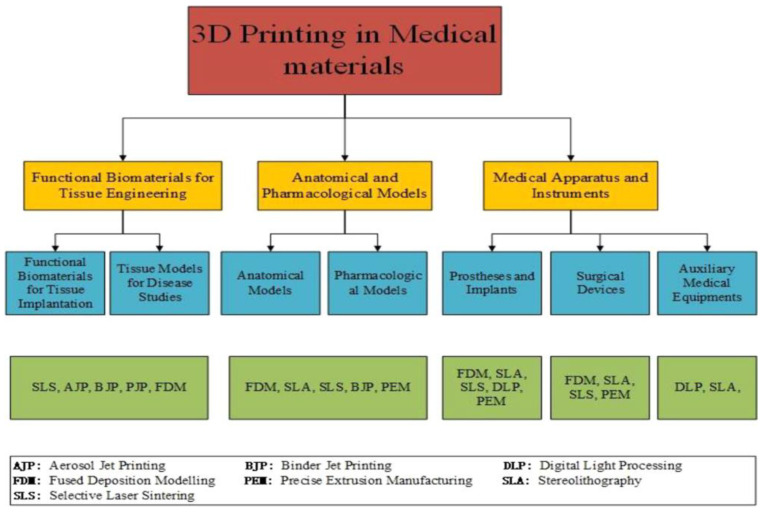
Progressive 3D printing technology and its application in medical materials. Chart showing the application area (yellow boxes), with corresponding products (blue boxes) and primary 3D printing techniques (green boxes) (Reprinted with permission from [105]).

Although there have been many advancements in applying polymer 3D printing research to medical applications, there are still numerous obstacles to overcome. Material characteristics are controlled by design performance, and process parameters are based on fabrication consistency [104]. Figure 10 shows the progressive 3D printing technology and its application in medical materials. Chart showing the application area (yellow boxes), with corresponding products (blue boxes) and primary 3D printing techniques (green boxes). Improving 3D-printed polymer performance in medical applications requires the development of innovative, computational methodologies and integrated design methods that holistically incorporate process, design, and materials in the new product creation.

Although several 3D-printable polymer materials have been produced in recent years, it is not always easy to select materials for particular purposes. Material selection is crucial to generating a print with acceptable characteristics, but it is difficult since 3D-printed polymers have performance uncertainties and ranges that are dependent on the printing method and factors.

### Challenges and Limitations in Additive Manufacturing of Composite Materials

As discussed in previous sections, additive manufacturing has several advantages in the development of metal- and polymer-based composites. However, there are several challenges and limitations that prevent its widespread application [106]. The current challenges and limitations in composite additive manufacturing.

The need for specific properties such as printability, compatibility with the printing process, and post-processing requirements makes selecting appropriate composite materials for additive manufacturing difficult. Composite materials are frequently composed of multiple components, such as reinforcing fibers and matrix materials, and have complex formulations. Due to the unique rheological properties, viscosity, and fiber alignment of composite materials, achieving consistent and reliable printing parameters could be difficult. It is critical to optimize the printing process parameters for each specific composite material [107]. Furthermore, in composite materials, achieving desired fiber orientation and alignment is critical to ensuring desired mechanical properties. Additive manufacturing techniques struggle to precisely control fiber orientation during the printing process [108]. Anisotropic mechanical properties can result from random fiber orientation or misalignment, reducing the overall strength and performance of the printed parts.

Furthermore, proper reinforcing fiber distribution within the matrix is critical for optimal mechanical properties. Fibers are typically discontinuous and randomly distributed in some additive manufacturing processes, such as fused filament fabrication. This can result in uneven mechanical properties and decreased overall strength of the composites. To achieve the desired mechanical properties and surface finish of composite parts, post-processing steps such as curing, heat treatment, and machining may be required. These extra steps can add complexity, time, and cost to the manufacturing process. It is difficult to develop efficient post-processing techniques for additive composite manufacturing [109]. Composite additive manufacturing is frequently limited in terms of scale and production rate. While it excels at producing complex, low-volume parts, due to the time required for printing and post-processing, the process may not be as efficient for large-scale production. The challenge of scaling up the process while maintaining consistent quality and productivity must be addressed. It is critical to ensure consistent quality and repeatability of composite parts produced through additive manufacturing, especially in industries with stringent regulations and standards. Creating standardized testing methods, quality control protocols, and certification processes for additive composite manufacturing is an ongoing challenge.

To address these challenges and limitations, ongoing research, the development of new materials, process optimization, and advancements in additive manufacturing technologies will be required. If these obstacles are overcome, the additive manufacturing of composite materials has the potential to revolutionize industries such as aerospace, automotive, and healthcare [110].

## 7. Conclusions

In this review paper, using recent results, an overview of AM of polymeric composites has been carried out. The use of AM concepts creates a huge impact on the development of composite materials with different shapes and complex structures.

1. Among all combinations available, the polymer-based materials were found to be excellent, which suits the 3D printing technology. The development of 3D printing technology is rapid. Numerous published publications and mechanical components in the biomedical, aeronautics, electronics, architectural, building fields, and automotive industries confirm this progress.
2. The commercially available natural fibers like hemp, flax, and wool are used to produce composite materials which act as a better replacement for synthetic materials which are also effective in the AM technique.
3. Polymeric materials such as PLA, PP, ABS, PEEK, and PC possess very good mechanical properties and are predominantly used to produce high-performance structural parts in aerospace and marine applications.
4. AM techniques are also preferred for metal-based fabrication, which has higher supportive products for thermal and high-temperature applications. In medical and biotechnology fields, the utilization of the AM technique is found more in which they are used to produce replacements for prosthetics, tissues, teeth, and bone implants.
5. Additive manufacturing technology creates an impact in the field of sustainable manufacturing with a wide range of applications and products with the introduction of newer materials that offer different properties through manufacturing in the markets.

## Figures and Tables

**Table 1:** Characteristics of polymer-based AM techniques.

Method	Advantages	Disadvantages	Applications
SLA	It has the capability of larger sizes. The final material is a flexible and transparent one	The material possesses a brittle property which makes it not suitable for mechanical component	Medical and jewel-making fields
SLS	It has better mechanical properties and is also able to machine huge parts	The cost is higher than that of FDM. The lead time consumption is also high.	Polymer-based components
FDM	This is preferred due to its low cost. For RPT this one is preferred	The dimensional accuracy is medium only, and the printing speed is very low	Casting and electrical appliances
Material Jetting	This possesses resistance to high temperatures. Also, better surface finish and accuracy.	High cost compared to SLA also has brittle behavior that is not suitable for mechanical applications	Injection-molded prototypes
LOM	This can machine large part sizes as well as large casting; the printing speed is high and has good tolerances; it is also more environmentally friendly	The surface finish is a concern to be addressed	Automotive sectors

**Table 2:** Characteristics of metal-based AM techniques.

**Method**	**Advantages**	**Disadvantages**	**Applications**
SLM	It has the capability of machining complex geometries.	Among all the available technologies, it is the most expensive one.	Used in the biomedical, aerospace, and automotive sectors.
Binder Jetting	Provides a high-quality surface finish with good precision.	The processing speed is limited, as the well as mechanical properties are not good compared to others.	Casting and architecture.
LENS	All of these techniques possess excellent metallurgical properties. It is also used in part for reconditioning.	Possess a lack of supporting structures due to different materials usage	Medical sectors and turbines.

## Data Availability

Not applicable.
